# A Study on Evaluation of Apoptosis and Expression of Bcl-2-Related Marker in Wound Healing of Streptozotocin-Induced Diabetic Rats

**DOI:** 10.1155/2013/739054

**Published:** 2013-10-07

**Authors:** Surya Bhan, Rahul Mitra, A. K. Arya, H. P. Pandey, K. Tripathi

**Affiliations:** ^1^Department of Biochemistry, School of Life Sciences, NEHU, Shillong 793022, India; ^2^Department of Biochemistry, Institute of Medical Sciences, Banaras Hindu University, Varanasi 221005, India; ^3^Department of Medicine, Institute of Medical Sciences, Banaras Hindu University, Varanasi 221005, India; ^4^Department of Biochemistry, Faculty of Science, Banaras Hindu University, Varanasi 221005, India

## Abstract

Uncontrolled blood sugar is a major cause of vascular complications and delayed wound healing in diabetes mellitus. During wound healing process, normally, apoptosis is responsible for events such as removal of inflammatory cells and evolution of granulation tissue into scar which occur during the late phase of wound healing. Early apoptosis can lead to abnormal wound healing by removing granulation tissue including fibroblast, endothelial cell, and small vessels. To determine the role of apoptosis in association with hyperglycemia in diabetic wound healing, apoptosis-related intracellular marker such as expression of Bcl-2 protein by immunohistochemistry and normal histology has been studied. Histological findings show higher level of apoptosis and diminished granulation tissue formation in diabetic rats wounds along with minimal expression of Bcl-2 in diabetic rats wounds when compared with nondiabetic rats wounds. It can be concluded from this study that elevated blood sugar level may be associated with increased apoptosis and the least expression of Bcl-2 protein which might cause deregulation of the wound healing processes in streptozotocin-induced diabetic rats.

## 1. Introduction

Apoptosis is a physiological cell death process, possesses very distinct features, and is almost different from necrosis which is characterized by morphological changes, including DNA fragmentation, membrane alteration, and formation of apoptotic bodies. It is one of the physiological cell death processes, which governs developmental biology and cellular homeostasis in an organism. There is enormous kind of evidence that the sequence of cellular events that characterize healing of cutaneous wound and other tissue repair processes is tightly regulated and controlled by a distinct temporal pattern of cellular apoptosis [1]. The three classically defined phases of wound healing, that is, inflammation, tissue formation, and tissue remodeling involve the differential participation of resident cells and infiltrating leukocyte subtypes [2]. After the initial haemostatic event, early wound repair is characterized by the invasion of neutrophils, macrophages, and lymphocytes, which serves as source of inflammatory and growth-promoting cytokines [3]. The fibroblasts migrate, proliferate, and synthesize extracellular matrix components, participating in the formation of granulation tissue. Cellular infiltration and proliferation must be sufficient and pronounced for normal early progression of wound repair. It has been discussed that this rapid increase in cell proliferation is allowed by an initial decrease of apoptosis. Later, when the inflammatory process begins to shut down with wound closure and scar evolution, there is a dramatic decrease of cellularity, which has been clearly shown to be mediated by an increase of apoptotic cell death [4, 5]. Conversely, when granulation tissue cells are not removed because of failing apoptosis, there are pathologic tissue repair and development of hypertrophic scar or keloid, both characterized by a high degree of cellularity [6]. Healing of wounds in diabetes is characterized by delays in the repair process as well as a decrease in the tensile strength of healing wounds. Hyperglycemia is major cause of vascular complications and delayed wound healing in diabetes mellitus. In diabetes accelerated disappearance of capillary endothelium, morphological and functional alterations of endothelial, fibroblast, inflammatory cells, and excess apoptosis have been reported. Several factors contribute to apoptosis, but the key elements are categorized in two main families of proteins including caspase enzymes and Bcl-2 family [7]. Bcl-2 is a protooncogene which is capable of inhibiting apoptosis or programmed cell death, and it encodes a 25 kDa protein, which can be found on inner mitochondrial membrane, nuclear envelope, and endoplasmic reticulum [8, 9]. Originally, Bcl-2 was found in follicular lymphomas where it was associated with a chromosomal t (14 : 18) translocation [10]. Such translocation leads to an overexpression of Bcl-2 leading to the inhibition of apoptosis which then contributes to neoplastic transformations [8, 11].

Immunohistochemical expression of Bcl-2 has been reported in several lymphatic, neurogenic, and epithelial neoplasms [12–14]. Alteration of apoptosis and cell proliferation have been implicated in wound healing process [15]. Expression of apoptosis-related marker such as Bcl-2 gives the new mean of regulating the apoptosis pattern in diabetic and nondiabetic wound healing of rat model of diabetes. To determine the role of apoptosis in association with hyperglycemia in diabetic wound healing, we have analyzed the expression of Bcl-2 protein, apoptosis, and normal histology in granulation tissue of streptozotocin-induced diabetic and nondiabetic rats which are involved in the removal of inflammatory cells during wound healing processes of diabetes.

## 2. Material and Method

### 2.1. Diabetes Induction

All animal procedures were performed with the approval of the Animal Ethical Use and Care Committee at the North Eastern Hill University, School of Life Sciences, Shillong, India. They were housed under ideal laboratory conditions, maintained on standard pellet diet, and watered *ad libitum *throughout the experimental period. Diabetes was induced in male albino rats (*n* = 10) weighing (100–150 g) with an injection of streptozotocin (Sigma) 70 mg/kg body weight (I.P) dissolved in citrate buffer pH 4.5. Noninjected rats (*n* = 10) were used as control.

### 2.2. Plasma Glucose Measurement

Four days after streptozotocin injection, the animals were tested for glucose in blood by using commercially available glucose estimation kit to confirm the diabetes. Animals having blood sugar level greater than 250 mg/dL were included in the study.

### 2.3. Wound, Preparation

After confirmation of diabetes, rats were anesthetized with ketamine (80 mg/kg body weight) intraperitoneally, and full-thickness excisional wounds were made on the back of the eight-week-old rats in 8 mm diameter. Biopsy specimens were obtained at the 5th, 10th, 20th, and 30th days after preparation of wounds and kept in 10% neutral buffered formalin for histology and immunohistochemistry.

### 2.4. Histology and Morphological Evaluation

Wound tissue was excised, ringed in cold phosphate buffer saline (PBS), and fixed in 10% neutral buffered formalin. After washing with distilled water, tissue was placed in dehydration process through graded ethanol. After two changes with xylene a block was prepared and cut into 5 mm thin sections by microtome machine. Sections were stained with hematoxylline and eosin, mounted, and finally observed under light microscope.

### 2.5. Biochemical Test for DNA Fragmentation

DNA fragmentation was studied by the methods described elsewhere [16]. It is based on the fragmentation of genomic DNA into larger and smaller fragments due to endonuclease activity during apoptosis and could be separated by centrifugation. Briefly wound tissue was rinsed in PBS weighted, preminced with fine scissors, and homogenized. The cells were washed with PBS, trypsinised, and centrifuged at 110 g for 5 minutes to remove trypsin. The cell pellets were lysed with 0.4 mL of hypotonic lysis buffer (10 mm Tris-HCl, 1 mm EDTA PH 7.5) containing 0.5% triton x-100, and lysate was centrifuged at 13000 ×g for 10 minutes to separate intact chromatin from fragmented DNA. The supernatant, containing fragmented DNA, was placed in separate microcentrifuge tube, and both pellet and supernatant were precipitated overnight at 4°C in 12.5% Trichloroacetic acid (TCA). The precipitates were sedimented at 13,000 g for 4 minutes. The DNA in pellets and supernatant was hydrolyzed by heating to 90°C for 10 minutes in 50 mL of 5% TCA and quantified by modified method of Burton. In this method 100 *μ*L of diphenylamine (DPA) reagent (150 mg DPA, 150 *μ*L sulphuric acid, 50 *μ*L acetaldehyde (16 mg/mL in glacial acetic acid)) was added to each tube. After overnight color development, 200 *μ*L of each sample was transferred to 96 wells of ELISA plates, and an optical density was measured at 570 nm in microplate reader. The percentage of DNA fragmentation was defined as a ratio of DNA in the 13,000 g supernatant to the total DNA in the 13,000 g supernatant and pellet.

### 2.6. Immunohistochemistry

Five *µ*m thick sections were cut from the specimens and placed on poly-L-lysine-coated slides. They were then dewaxed in xylene and rehydrated in graded alcohol. The endogenous peroxidase activity was consumed by immersing the sections in 0.1% hydrogen peroxide in absolute methanol for 20 minutes. Nonspecific binding was blocked by incubating the slides in 20% fetal calf serum in PBS for 20 minutes. A monoclonal antibody directed against Bcl-2 oncoprotein was obtained from Santa Cruz Biotechnology, USA. Before application of the primary antibody, the sections were heated in a microwave oven in 10 mM citric acid monohydrate, pH 6.0 for 3 minutes. After one hour of incubation with the primary antibody (dilution 1 : 50) a biotinylated secondary anti-mouse antibody was applied followed by the HRP-biotin-peroxidase complex. The color was developed by adding diaminobenzidine and mounted. Negative control was prepared by omitting the primary antibody. For positive control follicular lymphoma was used.

## 3. Results

### 3.1. Blood Glucose, Apoptotic Index, and DNA Fragmentation Evaluation

The mean blood sugar level in control group rats on the 5th, 10th, 20th, and 30th days was 75.62 ± 6.41, 80.79 ± 10.58, 92.05 ± 12.62, and 90.77 ± 9.7 mg/dL, respsctively, and in diabetic group rats it was 467.25 ± 48.2, 506.33 ± 35.89, 474.99 ± 39.76, and 488.15 ± 34.36 mg/dL, respectively (Table 1). The blood glucose level was maximum on the 20th day and minimum on the 5th day in control group. In diabetic group it was found to be maximum on the 10th day and minimum on the 5th day. There was significant difference in the mean of blood glucose of both groups (*P* < 0.01). The mean apoptotic index in control group rats on the 5th, 10th, 20th, and 30th days was 1.50 ± 0.60, 1.60 ± 0.99, 1.64 ± 0.86, and 1.70 ± 1.12, respectively (Table 1), and in diabetic group rats it was 3.50 ± 2.60, 4.20 ± 2.99, 3.60 ± 3.56, and 3.69 ± 2.75, respectively (Table 2). There was a significant difference in the mean of apoptotic index in control and diabetic groups (*P* < 0.01). The mean % DNA fragmentation in control group 42.25 ± 3.95, 44.15 ± 5.61, 45.45 ± 5.88, and 46.58 ± 5.95 (Table 1) and in diabetic group 62.80 ± 9.56, 74.95 ± 10.45, 66.55 ± 8.67, and 70.48 ± 6.21 (Table 2). The significant difference in the mean % DNA fragmentation of both groups (*P* < 0.01) was found. 

### 3.2. Light Microscopy Findings

**Figure 1:**
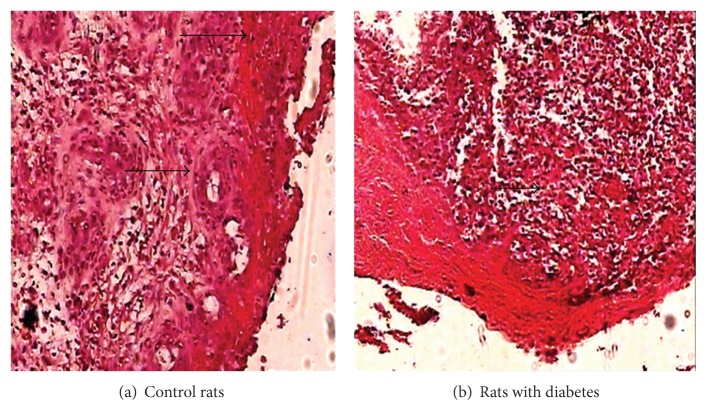
Histological findings in control rats and rats with diabetes. (a) The migration of inflammatory cells, formation of new blood vessels, and tissues architecture (indicated by arrow) in control rats and (b) diminished formation of granulation tissue, provisional matrix, and delayed healing in wounds of rats with diabetes (indicated by arrow) (H & E staining).

At the earliest time point provisional matrix with inflammatory cells and dilated blood vessels could be observed underneath a newly formed epithelial layer. Reepithelialization was completed after wounding. Maturation of granulation tissue was detected and characterized by the presence of new blood vessels, inflammatory cells, and collagen fibers organized into a dense connective tissue in control group (Figure 1(a)) on the 30th day. In case of diabetic rats wound showed the diminished formation of granulation tissues as the time advances almost on the 30th day which are necessary for the matrix formation (Figure 1(b)).

### 3.3. Apoptotic Morphology

**Figure 2:**
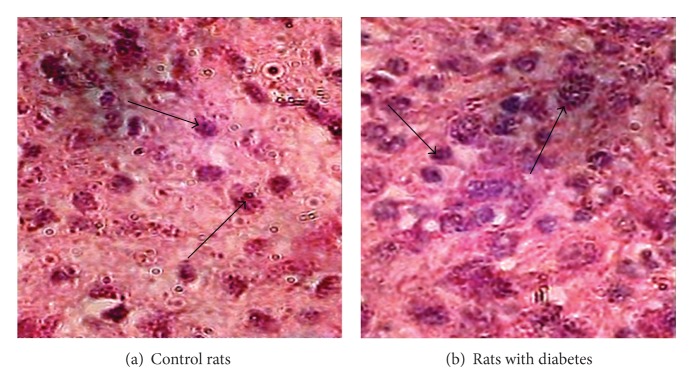
Distinct number of apoptotic cells were found and observed in both groups, but it was significantly higher in wounds of rats with diabetes (b) when compared with control group (a) (H & E staining) (arrow clearly showed the apoptotic cells characterized by nuclear condensation and fragmentation in both groups).

Apoptotic cells in control group and in diabetic rats wounds group were identified on the basis of morphological features that induced central cell apoptotic bodies, uniformly condensed chromatin, and densely stained nucleolus or membrane bound apoptotic bodies containing one or more nuclear fragments. The apoptotic cells were determined by counting more than 300 cells in at least three separate regions. Apoptotic cell death was monitored by morphological analysis that showed increased number of apoptotic cells in diabetic wound group on the 30th day (Figure 2). Less number of apoptotic cells was observed in control group.

### 3.4. Immunohistochemistry

**Figure 3:**
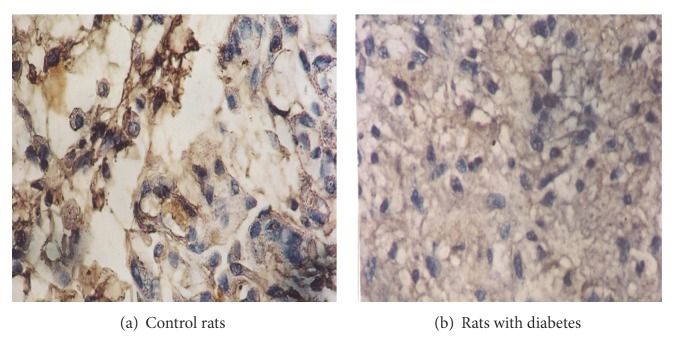
Immunohistochemical localization and expression of the bcl-2 (antiapoptotic protein) in (a) control rats and (b) wounds of rats with diabetes.

The Bcl-2 staining and its expression as protooncogene were observed in granulation tissue of control and diabetic rats wounds on the 30th day. Bcl-2 staining and expression were positive and more intense in control rats wounds. In the case of diabetic rats wound, no Bcl-2 positive staining or Bcl-2 expression was observed as the time advances (Figure 3).

## 4. Discussion 

Wound healing processes can be divided mainly in three distinct stages: (i) fibroblast proliferation and secretion of mucopolysaccharides, (ii) replacement of mucopolysaccharides by collagen molecule and maturation of these molecules by cross-linking, and (iii) remodeling and scar formation. Cell proliferation, protein synthesis, and protein release are also evaluated in wound healing process. Healing of wounds in diabetes is characterized by delays in the repair process as well as the decrease in the tensile strength of healing wounds. Deficiencies in fibroblast numbers have been reported to represent an important aspect of delayed wound healing in diabetes. It has been suggested that aberrant growth factor expression, altered inflammatory responses, or enhanced glycosylation of proteins may be involved alternatively; enhanced apoptosis may decrease fibroblast numbers, which could contribute to the impaired diabetic healing. In this study we found that inflammatory cells and dilated blood vessels could be observed underneath a newly formed epithelial layer in nondiabetic rats. Maturation of granulation tissue was detected and characterized by the presence of new blood vessels, inflammatory cells, and collagen fibers organized into a dense connective tissue in control group (Figure 1(a)), but in case of diabetic rat wound showed the diminished formation of granulation tissues which are necessary for the matrix formation (Figure 1(b)). Whatever the cause is, the generation and maintenance of a sufficient number of fibroblasts to participate in wound repair may be particularly important in diabetes. Humans with type 1 diabetes have impaired cutaneous wound healing. This may be caused by reduced bone formation, as supported by findings that serum osteocalcin levels are significantly lower in type 1 diabetic patients, and there is a reduction in osteoblast numbers and function. In type 2 diabetes, there is evidence of diminished bone formation, increased risk of fracture, and impaired healing, but the mechanisms are not well established.

Apoptosis has been identified as the mechanism of cell death in a number of degenerate diseases. Apoptosis is an active process that has described well biochemical and morphological characteristics including lack of inflammatory response and DNA fragmentation caused by endonuclease activity. It is identified in histological sections by pyknotic nuclei, cytoplasmic condensation, and DNA fragmentation which can be stained by in situ end labeling (TUNEL) of single or double strands break in DNA. The study reports that both experimental diabetes in rats and diabetes mellitus in humans are accompanied by increased apoptosis and biochemical changes in wound healing processes. It was found that inflammatory cells undergo apoptosis [17]. Our finding also indicates the similar results which showed the increased number of apoptotic cells during healing processes in diabetic wound. The cells which undergo the process of apoptosis are mainly inflammatory cells including neutrophils and lymphocytes as they have been observed under microscope in diabetic wound. During morphological study we found that there were significant number of apoptotic cells in diabetic wound compared with nondiabetic wound (Figure 2). Increased apoptosis in diabetic wound may be due to persistent hyperglycemia and many play an important role through oxidative stress mechanism, and further studies are needed to explore the signaling pathways. The effect of blood sugar level was evaluated by comparing the blood sugar level with DNA fragmentation and apoptotic index. Blood sugar is the main determinant of apoptosis and persists for longer time to play an important role in metabolism. This clearly explains our finding of an increased DNA fragmentation correlated with blood sugar (Tables 1 and 2). In comparative studies of full-thickness wounds in diabetic and nondiabetic mice, they observed that apoptosis was detected in the inflammatory cells as early as 12 hours in nondiabetic mice. The apoptosis level peaked at the fifth day after wounding and slowly decreased afterwards. Infiltration of inflammatory cells was present within 12 hours, consisting primarily of neutrophils. Apoptosis was consistently observed in the inflammatory cells beneath the leading edge of migrating epithelium. This may be indication that apoptosis signals the end of the inflammatory phase of healing. In comparison to nondiabetic mice, diabetic mice were characterized by delayed apoptosis, but the trend was reversed when topical PDGF and insulin-like growth factor II (IGF-II) treatment was applied to the wound by the another study. Desmouliere et al. investigated the role of apoptosis in the evolution of granulation tissue to scar tissue [18]. Using eight-week-old Wistar rats, granulation tissue was sampled from 2 to 60 days. They observed many apoptotic inflammatory cells during the first few days after wound healing. In particular, isolated apoptotic cells were visible by eight days, peaking from 12 to 25 days. Furthermore, the frequency of apoptotic myofibroblasts and vascular cells was highest during this time period. Their data also suggest that apoptosis of the granulation tissue begins after wound closure, affecting cells in a consecutive fashion. This concurs with the observation of gradual granulation tissue resorption after wound closure.

Apoptosis has also been implicated in dermal reconstruction. In a study by Rossio-Pasquier et al., athymic nude mice were grafted with split-thickness human skin biopsies [19]. Two months later, partial-thickness wounds were made, and the healing grafts were harvested from 1 to 4 days after injury. The same experiment was repeated using petrolatum-impregnated dressings. On day 1 after the injury, TUNEL-positive cells were present in the uninjured human dermis beneath the scab for mice treated without petrolatum-impregnated dressings. Similar observations were subsequently noted until day 4 when no apoptotic cells were seen. In contrast, for mice with petrolatum-impregnated dressings, very few TUNEL-positive cells were seen during the first three days after wounding, and no apoptotic cells were seen by day 4. The results suggest that human fibroblasts disappeared from the uninjured dermis beneath the scab via apoptosis and that petrolatum-impregnated dressings reduced the level of apoptosis.

Kane and Greenhalgh further the role of apoptosis-related markers during inflammation at the leading edge of the epithelium was explored [20]. They compared the expression of p53, Bcl-2, and apoptosis between normal and diabetic mice for 42 days. Their results suggest that normal mice exhibit an inverse relationship between Bcl-2 and p53 over time. Upon injury, Bcl-2 expression was increased, while that of p53 decreased, in order to allow cellular proliferation to occur. As the inflammation process declined, p53 levels decreased. The role of apoptosis appeared to parallel the rate of p53. For diabetic mice, there was no inverse relationship, and p53 expression was, consistently, higher than that of Bcl-2. In our study we found the same result where Bcl-2 was little or very less expressed in diabetic rats on the 30th day of wound healing. The results clearly indicated the downregulation of a Bcl-2 protein in wound healing of diabetic rats. The levels of both p53 and Bcl-2 decreased over the days from 21 to 42, and the peak of apoptosis did not occur until day 14. Although the relationship of these apoptosis markers is not well understood in diabetic mice, this study illustrates the necessary balance between p53 and Bcl-2 in normal mice during the inflammatory phase of wound healing. In conclusion we would like to add that in diabetic rats there were increased apoptosis and less expression of Bcl-2 protein. Further studies are required to explore the mechanism of increased apoptosis and relationship with Bcl-2 protein.

## Figures and Tables

**Table 1:** Mean blood glucose level, apoptotic index, and DNA fragmentation in control rats.

	5th day	10th day	20th day	30th day
Control (*n* = 10) Blood glucose (mg/dL)	75.62 ± 6.41	80.79 ± 11.45	92.05 ± 9.56	90.77 ± 9.7
Apoptotic index (Mean ± Sd)	1.50 ± 0.60	1.60 ± 0.99	1.64 ± 0.86	1.69 ± 1.12
DNA fragmentation (%) (Mean ± Sd)	42.25 ± 3.95	44.15 ± 5.61	45.45 ± 5.88	46.58 ± 5.95

*P* value <0.01.

**Table 2:** Mean blood glucose level, apoptotic index, and DNA fragmentation in rats with diabetes.

	5th day	10th day	20th day	30th day
With diabetes (*n* = 10) Blood glucose (mg/dL)	467.25 ± 48.2	506.33 ± 35.89	474.99 ± 39.76	488.15 ± 34.36
Apoptotic index (Mean ± Sd)	3.50 ± 2.60	4.20 ± 2.99	3.60 ± 3.56	3.69 ± 2.75
DNA fragmentation (Mean ± Sd)	62.80 ± 9.56	74.95 ± 10.45	66.55 ± 8.67	70.48 ± 6.21

*P* value <0.01.
